# Randomised clinical trial for morphological changes of trabecular meshwork between Kahook dual-blade goniotomy and ab interno trabeculotomy with a microhook

**DOI:** 10.1038/s41598-023-48121-5

**Published:** 2023-11-27

**Authors:** Shogo Arimura, Kentaro Iwasaki, Yusuke Orii, Ryohei Komori, Yoshihiro Takamura, Masaru Inatani

**Affiliations:** Department of Ophthalmology, Faculty of Medical Sciences, University of Fukui, 23-3 Simoaizuki, Matsuoka, Eiheiji, Yoshida, Fukui Japan

**Keywords:** Diseases, Eye diseases, Ocular hypertension, Glaucoma

## Abstract

We demonstrated whether the difference of trabecular meshwork remodeling occur depending on the incisional cross-sectional area by comparing Kahook dual-blade goniotomy (KDB) and ab interno trabeculotomy with a microhook. Phakic eyes with primary open-angle or exfoliative glaucoma were randomised into a KDB or a microhook group. The primary outcome was an incisional cross-sectional area quantified by anterior segment optical coherence tomography. In subgroup analysis, the number of patients with the unidentifiable incisional area was compared between the groups. Secondary outcomes were the rate of intraocular pressure changes, the laser flare metre values, corneal endothelial cell densities, the number of glaucoma medications, the usage rate per glaucoma medication type and postoperative complications between the two groups. A total of 29 eyes in 29 patients in the KDB and microhook group were included respectively, with an overall mean age of 72.6 ± 8.1 years. The incisional cross-sectional area of the KDB group was significantly larger at 1 week and at 1, 6 and 12 months (*p* < 0.01) postoperatively. The number of patients with the nonidentified incisional area was higher at 1, 6 and 12 months postoperatively (*p* ≤ 0.03) in the microhook group. The flare values in the KDB group were higher than those in the microhook group at 12 months postoperatively (*p* = 0.02). No significant differences were observed in other secondary outcomes. Incisional cross-sectional area remains larger in eyes treated with KDB goniotomy than in those treated with ab interno trabeculotomy with the microhook, whereas KDB goniotomy did not have an advantage in controlling intraocular pressure postoperatively.

**Trial registration:** UMIN000041290 (UMIN, University Hospital Medical Information Network Clinical Trials Registry of Japan; date of access and registration, 03/08/2020).

## Introduction

Glaucoma is the leading cause of blindness worldwide^1^. The main target of treatment for glaucoma is lowering the intraocular pressure (IOP) to slow down the progression. Surgical options are chosen when medications are not sufficiently effective. Minimally invasive glaucoma surgery (MIGS) has been increasingly well known due to its safety profile, rapid recovery and simple technique^2–5^. MIGS is a surgical procedure using an ab interno approach, very little or no scleral dissection and minimal or no conjunctival incision. Surgical options for MIGS are based on the target of trabecular meshwork (TM)^6–8^, suprachoroidal^9^ or subconjunctival space^10^. Representative MIGS focusing on TM incision in Japan are Kahook dual-blade (KDB, New World Medical, Inc, CA, US) goniotomy and ab interno trabeculotomy with a microhook (Inami, Co., Ltd, Bunkyo, Tokyo) to improve trabecular outflow through Schlemm’s canal. KDB goniotomy^3^ excises TM in a band^11^ with the blades. An ab interno trabeculotomy with the microhook^12^ is performed by making a TM incision with a line. The two devices have different tip shapes to improve trabecular outflow resistance (Supplementary Fig. S1). Therefore, postoperative TM structures are expected to be different between the two surgeries. A previous study^13^ using human eyes receiving laser trabeculoplasty showed increased TM cell reproduction and migration to repopulate the damaged TM, possibly due to laser stimuli. However, the repairing process of TM after an incision is not clearly known. Anterior segment optical coherence tomography (AS-OCT) is a non-invasive, noncontact imaging device used to obtain images of the anterior segment of the eye. AS-OCT provides information regarding the TM thickness and width as previously reported^14,15^. We prospectively compared the postoperative cross-sectional incisional areas in TM between KDB goniotomy and ab interno trabeculotomy with the microhook using AS-OCT for 1 year, focusing for the TM morphology after the two types of surgeries.

## Results

### Patient recruitment

**Figure 1:**
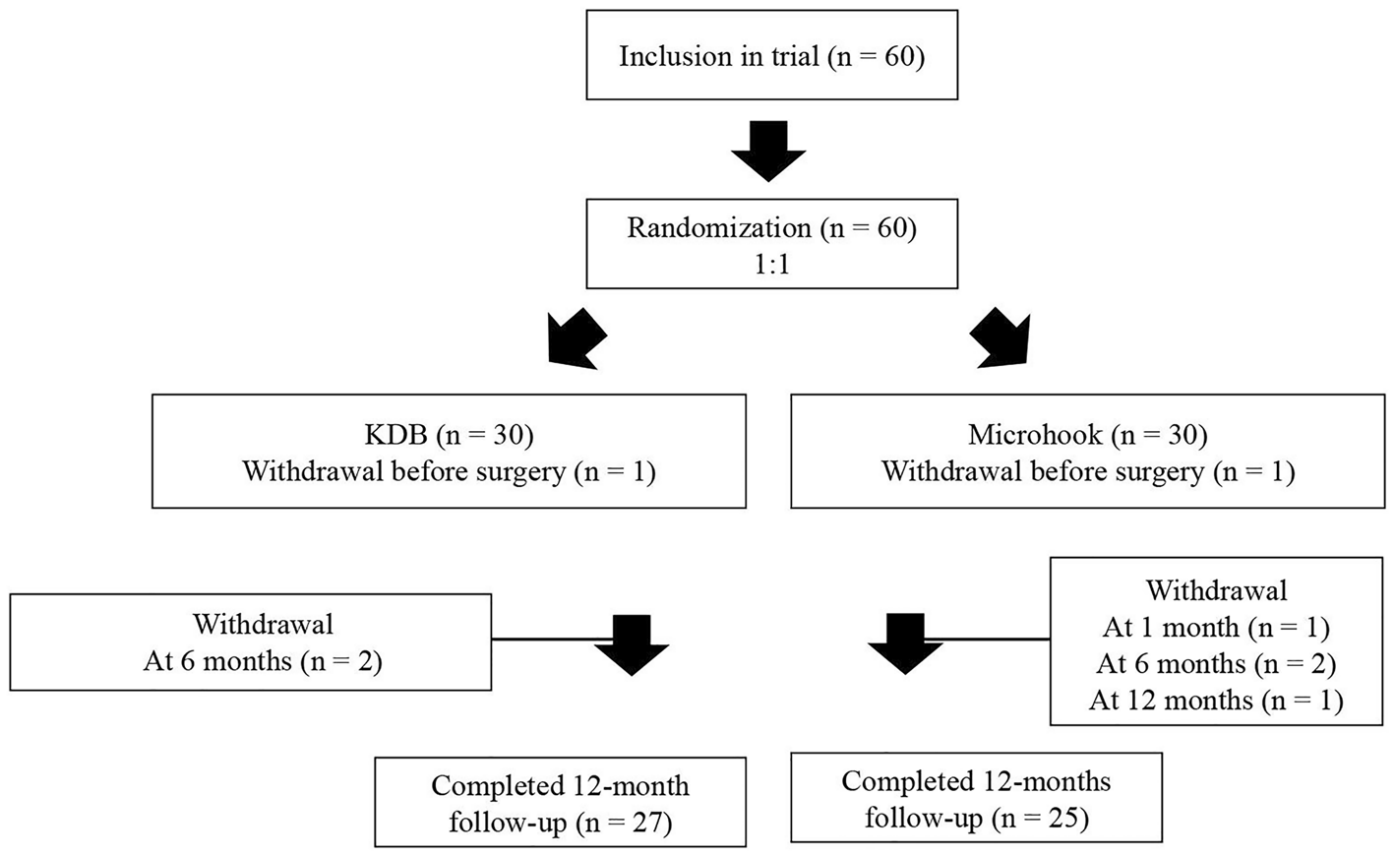
Diagram of the study design. Patients who met the inclusion criteria were randomised in a 1:1 ratio to receive KDB goniotomy or ab interno trabeculotomy with the microhook. After randomisation, one patient in each group was excluded due to the refusal to provide consent. A total of 27 and 25 patients completed the follow-up at 12 months in the KDB and microhook groups, respectively.

Figure 1 summarises patient recruitment. A total of 60 patients were recruited in this study. All patients were recruited from July 30, 2019, to February 21, 2021, at Fukui University Hospital. Thirty patients were assigned to each arm. One patient withdrew consent for the present study after the group assignment due to unwillingness to participate. A total of 27 and 25 patients completed follow-up visits for 1 year in the KDB and microhook groups, respectively. Two patients in the KDB group and four patients in the microhook group were lost to follow-up due to withdrawal. Table 1 shows the baseline characteristics of the study patients. No significant differences in baseline characteristics were observed between the KDB and microhook groups. There were no unintended harms in each group during all follow-up visits.

**Table 1:** Baseline characteristics.

	KDB group (n = 29)	Microhook group (n = 29)	*p*-value
Age (years)	72.9 ± 8.2	72.3 ± 8.3	0.98
F/M (n)	16/13	19/10	0.59
POAG/EXG (n)	22/7	22/7	1.0
IOP (mm Hg, IQR)	18.0 (16.0–20.0)	17.0 (14.0–19.0)	0.33
LogMAR VA	0.45 ± 0.45	0.62 ± 0.54	0.21
Flare (pc/ms, IQR)	14.5 (9.6–24.0)	12.1 (8.7–25.5)	0.82
Corneal ECD (cell/mm^2^, IQR)	2457 (2132–2618)	2545 (2404–2667)	0.08
Cataract (NC, IQR)	3.0 (2.0–3.0)	3.0 (2.0–4.0)	0.2
Glaucoma stage			
Mild (%)	20.7	17.9	
Moderate (%)	34.5	32.1	1.0
Severe (%)	44.8	50.0	
Glaucoma medications (n, IQR)	2.0 (1.0–3.0)	2.0 (2.0–3.0)	0.45
The type of glaucoma medications			
Prostaglandin (%)	86.2	89.7	1.0
β-blocker (%)	48.3	69.0	0.11
CAI (%)	37.9	48.3	0.43
Brimonidine (%)	37.9	31.0	0.58
Ripasudil (%)	50.0	48.3	0.90

Parametrical data are shown as mean ± standard deviation. Non-parametrical data are shown as median (IQR, range from the first to the third quartile). Unpaired t-tests, Mann–Whitney U-test, Pearson's chi-square test with or without Bonferroni correction, Fisher’s exact test.

*KDB* Kahook dual blade, *n* number, *F* female, *M* male, *POAG* primary open-angle glaucoma, *EXG* exfoliative glaucoma, *IOP* intraocular pressure, *LogMAR* logarithm of the minimum angle of resolution, *VA* visual acuity, *pc/ms* photocount per milli second, *ECD* endothelial cell density, *NC* nuclear colour according to Emery–Little classification, *CAI* carbonic anhydrase inhibitor.

### Primary outcome

**Figure 2:**
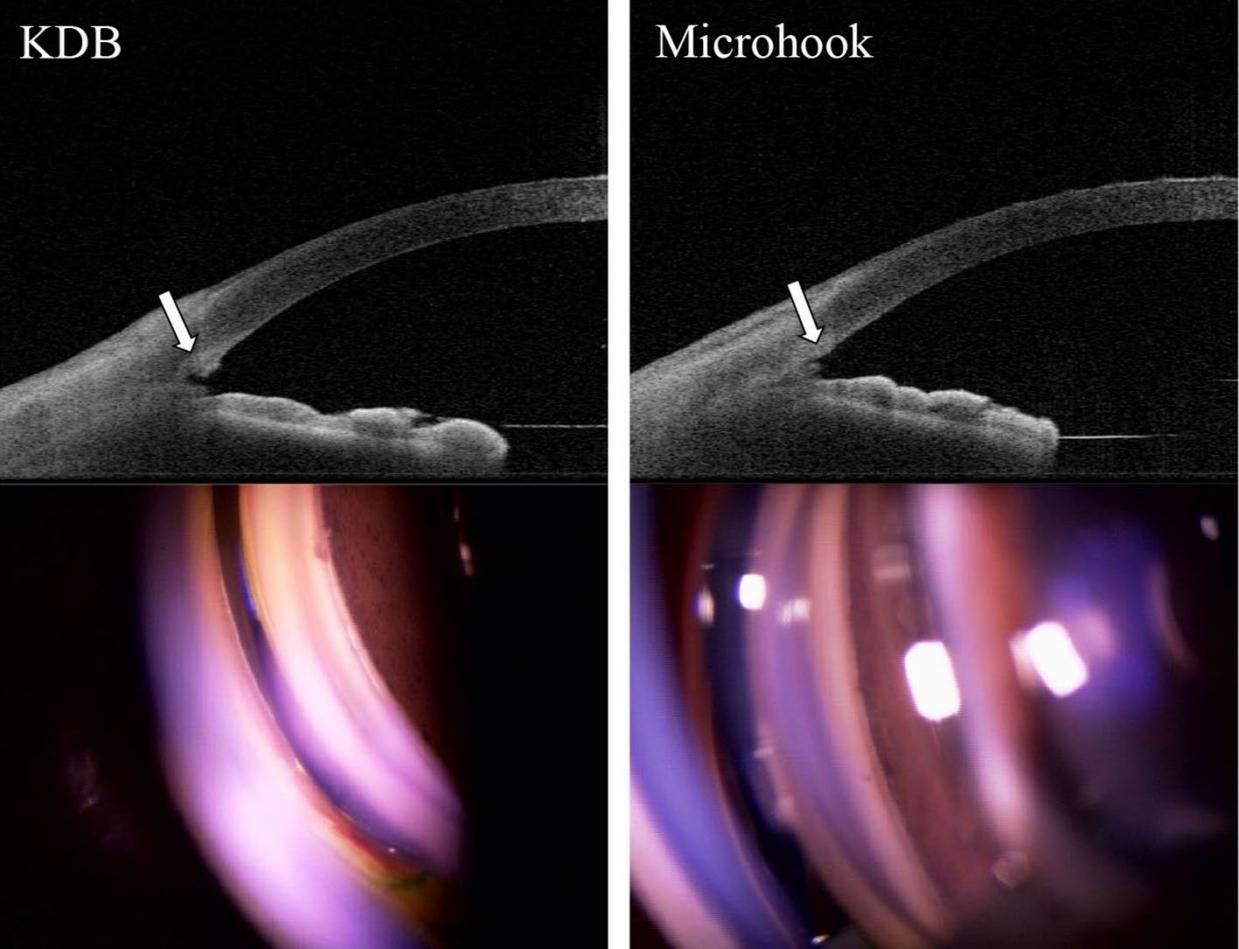
Representative images with AS-OCT and under gonioscopy after KDB goniotomy and ab interno trabeculotomy with the microhook. The arrow shows the incision sites. *AS-OCT* anterior segment optical coherence tomograohy, *KDB* Kahook dual blade.

**Figure 3:**
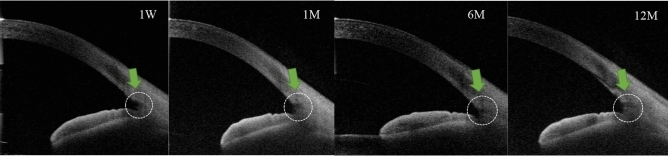
The time-dependent changes of the incisional site after an ab interno trabeculotomy with the microhook. The circle shows the incision sites. The incisional cross-sectional area was gradually smaller. In this case, the incision site cannot be completely identified at the 12-month follow-up. *1W* 1 week; *1M* 1 month, *6M* 6 months, *12M* 12 months.

The mean cross-sectional area of the incision sites in TM was larger in the KDB group during all follow-up visits (1 week; *p* < 0.01, 1 month; *p* < 0.01, 6 months; *p* < 0.01, 12 months; *p* < 0.01) (Table 2). Figure 2 shows representative images after KDB goniotomy and ab interno trabeculotomy with the microhook using AS-OCT and gonioscopy, respectively. As for the subgroup analysis, the number of patients with unidentified incisional cross-sectional areas with AS-OCT (area, 0) were zero in the KDB group and four in the microhook group at 1-week postoperatively (*p* = 0.05), which gradually increased during follow-up visits in both groups (Table 3). The number of patients with unidentified areas in the microhook group was significantly higher than that in the KDB group between the 1- and 12-month follow-up visits (1 month; *p* = 0.02, 6 months; *p* = 0.03, 12 months; *p* = 0.02). Figure 3 shows the representative eye that lost the cross-sectional area of the incision site in the microhook group during follow-up visits.

**Table 2:** The comparison of the incisional cross-sectional area (mm^2^) between the KDB and microhook groups.

	KDB group	Microhook group	*p*-value
1 week	3.2 × 10^–2^ (2.4 × 10^–2^ to 3.8 × 10^–2^)	1.1 × 10^–2^ (0.2 × 10^–2^ to 2.4 × 10^–2^)	< 0.01*
1 month	2.8 × 10^–2^ (1.6 × 10^–2^ to 3.6 × 10^–2^)	1.7 × 10^–2^ (1.5 × 10^–2^ to 2.2 × 10^–2^)	< 0.01*
6 months	2.6 × 10^–2^ (0.7 × 10^–2^ to 3.6 × 10^–2^)	1.2 × 10^–2^ (0–1.9 × 10^–2^)	< 0.01*
12 months	2.4 × 10^–2^ (0.7 × 10^–2^ to 3.5 × 10^–2^)	0.6 × 10^–2^ (0–1.9 × 10^–2^)	< 0.01*

Data are shown as median (IQR, range from the first to the third quartile). Mann–Whitney U-test.

*KDB* Kahook dual blade.

*Statistically significant, *p* < 0.05.

**Table 3:** Number of nonidentified cases (n) with AS-OCT and Image-J in the incisional sites between the KDB and microhook groups.

	KDB group	Microhook group	*p*-value
1 week	0	4	0.05
1 month	0	5	0.02*
6 months	3	11	0.03*
12 months	4	13	0.02*

The Fisher’s exact test.

*n* number, *KDB* Kahook dual blade.

*Statistically significant, *p* < 0.05.

The mean number of unidentified points of five due to PAS between the KDB group and the microhook group were 0.76 ± 0.12 and 0.72 ± 0.12, respectively (*p* = 0.77). There were no significant differences in cross-sectional area between the eyes with primary open angle glaucoma (POAG) and the eyes with exfoliative glaucoma (EXG) in all follow-up visits (Supplementary Table S1).

### Secondary outcomes

No significant differences in the rate of IOP changes were found between the two groups in all follow-up visits (Table 4). The 12-month postoperative flare values were significantly higher (*p* = 0.02) in the KDB group than that in the microhook group (Supplementary Table S2). No significant differences were observed in corneal ECDs and the number of glaucoma medications between the two groups (Supplementary Table S3 and Table 4). The usage rate per glaucoma medication type had no significant differences between the two groups postoperatively (Supplementary Table S4). Supplementary Table S5 showed the frequency of postoperative complications. There were no significant differences between the two groups.

**Table 4:** Postoperative rate of IOP changes (%) and number of glaucoma medications (n) between the KDB and microhook groups.

	The rate of IOP changes			Number of glaucoma medications		
	KDB group	Microhook group	*p*-value	KDB group	Microhook group	*p*-value
1 week	5.30 (− 1.70–25.0)	5.60 (− 1.80–30.8)	1.0	0 (0–0)	0 (0–1)	0.35
1 month	6.30 (0–21.1)	14.3 (0–20.9)	1.0	0 (0–1)	0 (0–1)	0.92
6 months	16.7 (8.20–25.0)	23.8 (9.70–33.3)	1.0	1 (0–2)	1 (0–2)	0.43
12 months	19.5 (11.8–24.9)	23.1 (7.00–38.3)	1.0	1 (0–2)	2 (0–3)	0.37

Data are shown as median (IQR, range from the first to the third quartile).

Mann–Whitney U-test with or without Bonferroni correction.

*KDB* Kahook dual blade, *IOP* intra ocular pressure, *n* number.

## Discussion

The purpose of the present study was to compare postoperative incisional cross-sectional area between KDB goniotomy and ab interno trabeculotomy with the microhook. The measurement using AS-OCT found a significantly larger incisional cross-sectional area in TM in the KDB group than in the microhook group throughout the postoperative follow-up visits (*p* < 0.01). The number of patients with unidentified incisional cross sectional areas by AS-OCT was significantly fewer in the KDB group than that in the microhook group between 1- and 12-months postoperative follow-up visits (1 month; *p* = 0.02, 6 months; *p* = 0.03, 12 months; *p* = 0.02). Our present data using AS-OCT revealed the difference in trabecular remodeling between the two MIGS procedures.

Several reports have focused on anterior chamber imaging after MIGS, the target for TM, including the anterior chamber parameter after trabectome^16,17^, iStent position after the surgeries^18,19^, and choroidal detachment after an ab interno trabeculotomy^20^, although no studies have examined the TM repairing process in human. Sequential imaging after surgeries in this study provides new insights, especially in the TM repairing process. Incisional cross-sectional areas after KDB goniotomy were significantly larger than that of ab interno trabeculotomy with microhook at all follow-up visits. The tip of KDB has two blades 230 µm wide in size, whereas the tip of the microhook is sharp without a blade. The two blades of KDB were cut through a TM, whereas the sharp tip of the microhook incises the TM. The different shapes of the tip could cause a difference in the incisional cross-sectional area between the two groups.

Subgroup analyses showed incisional cross-sectional areas in some patients appeared to be occluded immediately after the ab interno trabeculotomy with the microhook, and the number of patients continued to increase and was significantly higher than KDB goniotomy thereafter. No patients experienced obstruction immediately after KDB goniotomy; however, patients with occlusion gradually increased during follow-up visits. Only a few studies have been conducted on histological changes in TM after trabeculotomy using an animal model^21^. Ito et al. described the TM repair process after ab externo trabeculotomy in eight monkeys^22^. Electron microscopy data showed that the TMs of all patients were replaced by the repaired TM structure within a year postoperatively. The incisional site in 85% of patients in the KDB group was identified at postoperative 12-month follow-up visit in this study, while 48% of the microhook group was identified. KDB goniotomy is a technique that excises rather than incises TM with two blades. A large amount of excised volume by KDB goniotomy might contribute to attenuating the TM repair process.

Despite the significant difference in incisional cross-sectional area between the two groups, none of the rate of IOP changes, numbers of postoperative glaucoma medications and the usage rate per glaucoma medication type were found to show significant differences between the two groups. Postoperative IOPs of the two surgical procedures in our study are consistent with the IOP data in previous studies^23–25^. These results suggest that the efficacy of IOP reduction might not depend on the incisional cross-sectional area of TM postoperatively. The reason for no significant difference in the reduction of IOPs between the two groups has been unclear. Previous studies showed that electronic microscope observation for monkey eyes after *ab externo* trabeculotomy shows that the laminar structure of the TM after trabeculotomy appears to be replaced mainly by the corneoscleral meshwork and juxtacanalucular TM without the uveal meshwork, compared with the normal TM^22,26^. The structure after histological remodeling rather than the incisional cross-sectional area might be associated with the postoperative trabecular outflow facility.

This study showed that flare values were significantly higher in the KDB group at 12 months postoperatively. In general, the inflammatory phase is an essential process during wound healing^27^; however, chronic inflammation adversely affects wound healing. The repair process could be delayed in the KDB group due to chronic persistent inflammation in this study. The tip of KDB could be in contact with the iris intra-operatively due to the wider structure than that of microhook. Iris damage has been reported to cause higher inflammatory cytokine levels^28,29^ and chronic inflammation^30^ in the aqueous humour. Additionally, the plasma component from the episcleral vein might be provided into the anterior chamber in the KDB group because the incision site was maintained for a longer period after the KDB goniotomy.

Glaucoma medications, particularly prostaglandin and ripasudil, were related to inflammation. Prostaglandin can induce inflammation^31^ and ripasudil has anti-inflammatory properties^32^. In the present study, there were no significant differences in the usage rate of any type of glaucoma medications between the two groups. Besides, pseudo-exfoliation syndrome is known to be prone to postoperative inflammation^33^, which may affect remodeling in TM. In the present study, there were no significant differences in incisional cross-sectional area between the eyes with POAG and the eyes with EXG, postoperatively.

No significant differences were also observed in postoperative corneal ECDs between the two groups. Because the intra-operative incision site is close to the corneal endothelium, the tip structure of KDB might be associated with a higher risk of contracting the corneal endothelium intra-operatively. However, the difference in the tip width between the two MIGS devices does not contribute to the loss of corneal ECDs.

This study has some limitations. This study included both patients with and without stable IOP control. The results may differ if the trial was performed only on patients with uncontrolled IOP control. The capacity of the AS-OCT is limited to capture the size. Image-J was also used as an adjunct to evaluate the incisional cross-sectional area, although completely confirming how the TM is repaired is impossible. An evaluation using electron microscopy and a histological demonstration are required to determine the TM repair process in more detail. Flare values do not indicate all inflammatory factors in the anterior chamber. Since the laser flare metre measures the protein concentration, another experimental procedure such as an enzyme-linked immunosorbent assay would help evaluate the changes of cytokines and other protein molecules in the aqueous humour.

In conclusion, the incisional cross-sectional area was larger in the KDB group for all periods than in the microhook group, although no time points for the significance in the rate of IOP changes were observed between the two groups. The microhook group had the higher number of patients with TM-obstructed cross-sectional area observed by AS-OCT postoperatively. Therefore, TM repair may be less likely completed in patients with larger incisional sizes.

## Methods

The present study was approved by the institutional review board of Fukui University Hospital, Fukui, Japan, and registered by the University Hospital Medical Information Network Clinical Trials Registry of Japan (identifier University Hospital Medical Information Network; UMIN000041290; date of access and registration, 03/08/2020). Enrolled patients were randomly assigned 1:1 to undergo either KDB goniotomy or ab interno trabeculotomy with the microhook. Allocation was concealed until intervention was assigned. Sequentially numbered containers were used to perform allocation concealment. Y.T. generated the block random allocation sequence using random function. Block size was set to every four patients. K.I. and R.K. enrolled patients and Y.O. assigned the interventions. Enrolled patients were determined using the following criteria: (1) eyes with POAG or EXG. For each patient, if both eyes met the inclusion criteria, the eyes with higher IOPs were selected for the study. Open-angle glaucoma was diagnosed by an gonioscope examination. EXG was diagnosed based on a detailed slit-lamp examination and a gonioscope with the assessment of the presence or absence of clinical signs of pseudo-exfoliation syndrome: fibrillary deposits on the anterior capsule, pupillary border, iridocorneal angle, zonules or corneal endothelium and Sampaolesi line. Glaucomatous optic nerve head appears with a cup/disc ratio of ≥ 0.7, and the visual field test shows the defects at least satisfying Anderson and Patella’s criteria^34^. We performed glaucoma staging for every patient. We used the mean deviation value (MD) of the Humphrey visual field test for glaucoma staging. Glaucoma stage was defined as follows^35^: mild, MD >  − 6 dB; moderate, − 6 dB > MD >  − 12 dB; severe, − 12 dB > MD; (2) Japanese patients with cataract; and (3) minimum age of 20 years. The exclusion criteria were as follows: (1) history of ocular surgery and (2) pregnant, lactating, possibly pregnant and those wishing to become pregnant women during the study period.

### Surgical procedures

KDB goniotomy and ab interno trabeculotomy with the microhook were performed as per the instructions for use. Cohesive viscoelastic was injected into the anterior chamber through a 2.4-mm corneal incision temporally after the standard phacoemulsification and intraocular lens implantation. The patient’s head was rotated 30° to the contralateral side of the corneal incision area, and a surgical microscope is adjusted to allow nasal angle visualisation using the Ocular Hill Open Access Surgical Gonio lens (Ocular instruments, WA, US). Either the KDB or the microhook was inserted into the anterior chamber under the gonio prism. In KDB goniotomy, a strip of TM spanning for approximately 4 h was excised circumferentially in a band, whereas in ab interno trabeculotomy with the microhook, approximately 4 o’clock circumferential incision was created. All patients received similar preoperative topical medications: 0.5% levofloxacin three times daily for 3 days pre-operatively. All patients received similar postoperative topical medications: 0.5% levofloxacin and 0.1% betamethasone three times daily for a week and nepafenac three times daily for 3 months postoperatively, respectively.

### Primary outcome

**Figure 4:**
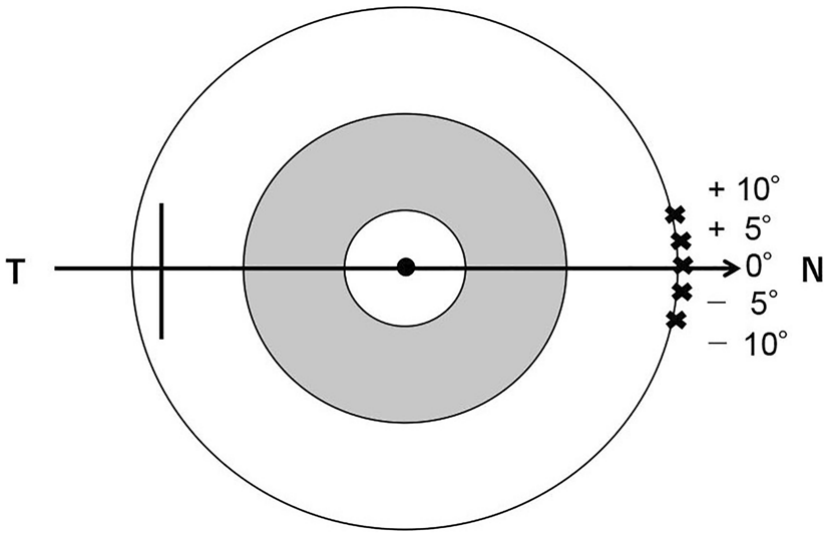
Five measurement points of an incision with AS-OCT. The exact contralateral trabecular meshwork of the corneal incision area was set at 0°, and measurement points were set at 5° increments (five points: − 10°, − 5°, 0°, 5° and 10°). The average of five measurement points was used in the analysis. *N* nasal, *T* temporal.

Follow-up visits were conducted at 1 week and 1, 6 and 12 months. The primary outcome was the incisional cross-sectional area in TM that was quantified with AS-OCT (CASIA, Tomey, Aichi, Japan). The exact contralateral TM of the corneal incision area was set at 0°, and measurement points were set at 5° increments (five points; − 10°, − 5°, 0°, 5° and 10°) as shown in Fig. 4. AS-OCT is equipped with a function to measure the incisional cross-sectional area. Image-J^36^ was used to measure the cross-sectional area using anterior images captured by AS-OCT only when the measurement point area cannot be identified with AS-OCT. As for the subgroup analysis, (1) the number of patients with the unidentifiable incisional site was compared (defined as an average of 5 measurement area, 0) with both AS-OCT and Image-J between the two groups. (2) We observed peripheral anterior synechia (PAS) formation under gonioscopy at least once over the entire observation period, postoperatively. The number in 5 measurement points that could not be identified because of PAS in the postoperative incisional cross-sectional area were compared between the two groups. (3) Besides, we compared the postoperative incisional cross-sectional areas in TM between the eyes with POAG and the eyes with EXG using AS-OCT in all follow-up visits.

### Secondary outcomes

Secondary outcomes were the rate of IOP changes from the preoperative IOPs, flare values, corneal ECDs, the number of glaucoma medications, the usage rate per glaucoma medication type, and postoperative complications. IOPs were measured by Goldmann applanation tonometry. The preoperative IOP was the average IOPs taken within 2 months pre-operatively. Anterior chamber inflammation was quantified as a flare value using a flare metre (FM-600, Kowa, Tokyo, Japan). The mean corneal ECD of each eye was measured using a noncontact specular microscope (NSP-9900 II, Konan, Nishinomiya, Japan) in five regions (central, superior, inferior, nasal and temporal). The type of glaucoma medications patients used preoperatively or postoperatively were prostaglandin, β-blocker, carbonic anhydrase inhibitor, brimonidine tartrate and ripasudil. The postoperative complications included frequency of hyphema, transient IOP elevation, macular edema and ciliochoroidal detachment. Transient IOP elevation was defined as an increase in IOP of 10 mmHg or more from baseline within 1 month postoperatively. Hyphema size larger than 1 mm was considered significant^35^.

### Sample size

The sample size was determined by the results of the preliminary investigation before this RCT. We hypothesized that the cross-sectional area in the KDB group would be higher than the microhook group. The sample size that would provide 80% power to prove the superiority of the KDB group for the incisional cross-sectional area was 25 eyes for each group with an effect size of 0.83. Therefore, we planned to recruit 60 eyes to account for a 15% dropout rate.

### Statistical analysis

Data were analysed using the SPSS version 27.0 (IBM Institute, Inc. Chicago, IL, USA). In each group, normality was measured with a Shapiro–Wilk test. Pearson's chi-square test or Fisher’s exact test was performed for categorical data. Unpaired t-tests or Mann–Whitney U-test with or without Bonferroni correction were performed for normally and nonnormally distributed data, respectively. For all tests, a *p*-value of < 0.05 was considered significant.

### A statement of ethics

The protocol adhered to the tenets of the Declaration of Helsinki. Written informed consent was obtained from all patients.

### Supplementary Information

Supplementary Figure 1.

Supplementary Tables.

Supplementary Information.

## Data Availability

Correspondence and requests for materials should be addressed to M.I.
