# Supplementary figures and images for: Randomised clinical trial for morphological changes of trabecular meshwork between Kahook dual-blade goniotomy and ab interno trabeculotomy with a microhook
Source: Sci Rep. 2023 Nov 27;13:20783. doi: 10.1038/s41598-023-48121-5 (PMC10682418; doi:10.1038/s41598-023-48121-5)

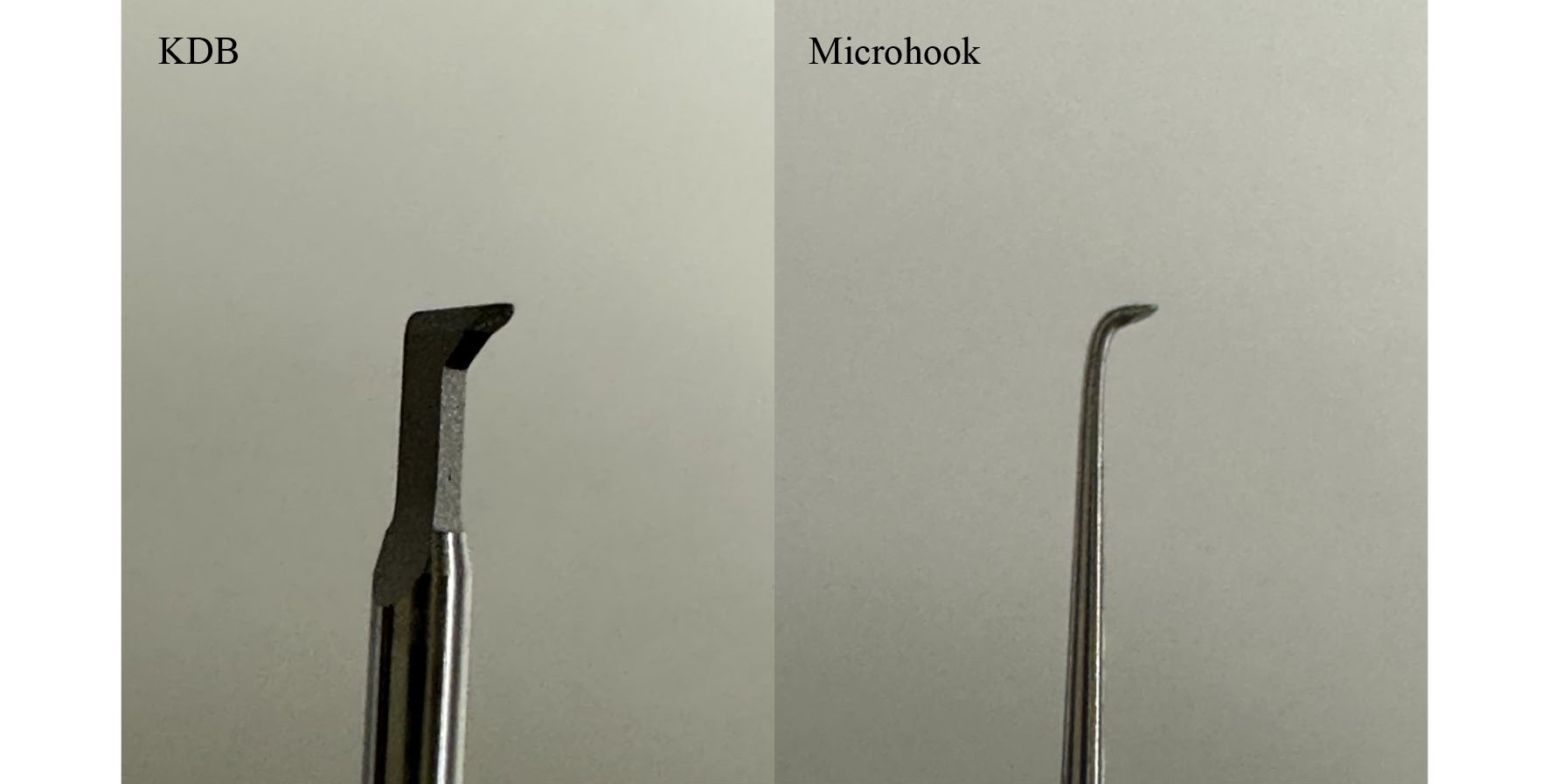

Supplement: Supplementary file 1 — Supplementary Figure 1. [file 41598_2023_48121_MOESM1_ESM.jpg]
