# Supplementary material for: Randomised clinical trial for morphological changes of trabecular meshwork between Kahook dual-blade goniotomy and ab interno trabeculotomy with a microhook
Source: Sci Rep. 2023 Nov 27;13:20783. doi: 10.1038/s41598-023-48121-5 (PMC10682418; doi:10.1038/s41598-023-48121-5)
Supplement: Supplementary file 2 — Supplementary Tables. [file 41598_2023_48121_MOESM2_ESM.docx]

**Supplementary Table S1.** The comparison of the incisional cross-sectional area (mm^2^) between POAG and EXG.

|  | POAG | EXG | *p*-value |
| --- | --- | --- | --- |
| 1 week | 2.4・10^-2^ (0.6・10^-2^-3.3・10^-2^) | 2.5・10^-2^ (0.7・10^-2^–3.4・10^-2^) | 0.83 |
| 1 month | 2.2・10^-2^ (0.6・10^-2^-3.1・10^-2^) | 2.5・10^-2^ (0.8・10^-2^–3.2・10^-2^) | 0.45 |
| 6 months | 1.8・10^-2^ (0-2.7・10^-2^) | 1.7・10^-2^ (0.6・10^-2^–2.5・10^-2^) | 0.68 |
| 12 months | 1.5・10^-2^ (0-2.7・10^-2^) | 1.7・10^-2^ (0.5・10^-2^–2.4・10^-2^) | 0.98 |

POAG, primary open angle glaucoma. EXG, exfoliative glaucoma.

Data are shown as median (IQR, range from the first to the third quartile).

Mann–Whitney U-test.

**Supplementary Table S2.** Postoperative flare values (pc/ms) of the KDB and microhook groups

|  | KDB group | Microhook group | *p*-value |
| --- | --- | --- | --- |
| 1 week | 22.9 (12.9–37.2) | 18.3 (10.9–26.3) | 0.10 |
| 1 month | 13.5 (9.50–23.3) | 12.5 (8.10–18.7) | 0.47 |
| 6 months | 11.4 (6.70–15.4) | 10.2 (6.40–14.4) | 0.70 |
| 12 months | 12.9 (8.30–21.3) | 8.8 (5.98–11.4) | 0.020* |

pc/ms, photocount/millisecond; KDB, Kahook dual blade.

Data are shown as median (IQR, range from the first to the third quartile).

Mann–Whitney U-test. * Statistically significant, *p* < 0.05

**Supplementary Table S3.** Corneal endothelial cell densities (cells/mm^2^) postoperatively between the KDB and microhook groups

|  | KDB group | Microhook group | *p*-value |
| --- | --- | --- | --- |
| 1 week | 2,263 (2,097–2,564) | 2,310 (2,005–2,540) | 0.98 |
| 1 month | 2,242 (2,004–2,427) | 2,307 (2055–2,494) | 0.80 |
| 6 months | 2,360 (2,116–2,545) | 2,373 (2,153–2,510) | 0.99 |
| 12 months | 2,304 (2,032–2,516) | 2,404 (2,285–2,543) | 0.22 |

KDB, Kahook dual blade.

Data are shown as median (IQR; range from the first to the third quartile).

Mann–Whitney U-test.

**Supplementary Table S4.** The usage rate per glaucoma medication type (%) postoperatively between the KDB and microhook groups

|  | KDB group | Microhook group | *p*-value |
| --- | --- | --- | --- |
| PG  1 week  1 month  6 months  12 months | 20.7  35.7  48.1  63.0 | 20.7  25.0  50.0  64.0 | 1.0  0.28  0.89  0.95 |
| β-blocker  1 week  1 month  6 months  12 months | 6.90  10.7  29.6  37.0 | 17.2  21.4  34.6  52.0 | 0.23  0.23  0.70  0.21 |
| CAI  1 week  1 month  6 months  12 months | 6.90  3.60  18.5  22.2 | 3.40  7.10  19.2  28.0 | 0.55  0.55  0.95  0.63 |
| Brimonidine  1 week  1 month  6 months  12 months | 10.7  0  18.5  22.2 | 20.7  10.7  30.8  48.0 | 0.25  0.12  0.24  0.080 |
| Ripasudil  1 week  1 month  6 months  12 months | 10.3  3.60  14.8  18.5 | 3.40  3.60  7.70  16.0 | 0.30  1.0  0.35  0.81 |

KDB, Kahook dual blade; PG, prostaglandin; CAI, carbonic anhydrase inhibitor.

Pearson's chi-square test, Fisher’s exact test.

**Supplementary Table S5.** Postoperative complications

|  | KDB group (n = 29) | Microhook group (n = 29) | *p*-value |
| --- | --- | --- | --- |
| Hyphema (%) | 17.2 | 10.3 | 0.35 |
| Transient IOP elevation (%) | 13.8 | 20.7 | 0.49 |
| Macular edema (%) | 0 | 3.40 | 0.50 |
| Choroidal detachment (%) | 0 | 0 | 1.0 |

KDB, Kahook dual blade; IOP, intraocular pressure

Pearson's chi-square test or Fisher’s exact test.
