# Supplementary material for: Randomised clinical trial for morphological changes of trabecular meshwork between Kahook dual-blade goniotomy and ab interno trabeculotomy with a microhook
Source: Sci Rep. 2023 Nov 27;13:20783. doi: 10.1038/s41598-023-48121-5 (PMC10682418; doi:10.1038/s41598-023-48121-5)
Supplement: Supplementary file 3 — Supplementary Information. [file 41598_2023_48121_MOESM3_ESM.docx]

**Supplementary Figure S1.** The image showing the differences in tip structure after Kahook dual blade goniotomy and ab inteno trabeculotomy with the microhook.

The Kahook dual blade has two blades to excise trabecular meshwork in a band. The microhook has no blade and making a trabecular meshwork incision with a line.

KDB, Kahook dual blade
